# Identification of prognostic biomarkers in a large cohort of patients with LGMD R2

**DOI:** 10.1007/s00415-026-13868-0

**Published:** 2026-05-26

**Authors:** Carla F. Bolano-Diaz, Jose Verdu-Diaz, Dan Hao, Meredith K. James, Laura Rufibach, Andrew Blamire, Harmen Reyngoudt, Pierre G. Carlier, Heather Gordish-Dressman, Heather Hilsden, Simone Spuler, John Day, Kristi J. Jones, Diana Bharucha-Goebel, Alan Pestronk, Maggie C. Walter, Carmen Paradas, Tanya Stojkovic, Madoka Mori-Yoshimura, Elena Bravver, Elena Pegoraro, Jerry Mendell, The Jain COS Consortium, Adrienne Arrieta, Marni Jacobs, Esther Hwang, Elaine Lee, Isabel Illa, Eduard Gallardo, Izaskun Belmonte Jimeno, Elena Montiel-Morillo, Irene Pedrosa-Hernández, Jaume Llauger Rossello, Bruce Harwick, Jackie Sykes, Susan Sparks, Scott Holsten, Lindsay Alfano, Megan Iammarino, Natalie Reash, Brent Yetter, Mark Smith, Emmanuelle Salort-Campana, Bernard Lapeyssonie, Bruno Vandevelde, David Bendahan, Yann Le Fur, Attarian Shahram, Testot-Ferry Albane, Eva M. Coppenrath, Sabine Krause, Olivia Schreiber-Katz, Simone Thiele, Ursula Moore, Michela Guglieri, Elizabeth Harris, Teresinha Evangelista, Alex Murphy, Michelle Eagle, Robert Muni Lofra, Anna Mayhew, Dionne Moat, Jassi Amritpal Singh Sodhi, Helen Sutherland, Tim Hodgson, Fiona E. Smith, Ian Wilson, Dorothy Wallace, Louise Ward, Debra Galley, Chiara Calore, Claudio Semplicini, Luca Bello, Roberto Stramare, Alessandro Rampado, Suna Turk, Ericky Caldas de Almeida Araujo, Noura Azzabou, Jean Yves Hogrel, Aurélie Canal, Cyrille Theis, Jean-Marc Boisserie, Julien Le Louër, Oumar Diabaté, Matthew Har, Julaine M. Florence, Catherine Siener, Linda Schimmoeller, Glenn Foster, Pilar Carbonell, Macarena Cabrera, Juan Bosco Mendez, Nieves Sánchez-Aguilera Práxedes, Yolanda Morgado, Susana Rico Gala, Jennifer Perez, Anne Marie Sawyer, Carolina Tesi-Rocha, Tina Duong, Richard Gee, Nigel F. Clarke, Sarah Sandaradura, Roula Ghaoui, Kayla Cornett, Claire Miller, Sheryl Foster, Anthony Peduto, Kristy Rose, Noriko Sato, Takeshi Tamaru, Shin’ich Takeda, Ai Ashida, Tatayuki Tateishi, Hiroyuki Yajima, Chikako Sakamoto, Takahiro Nakayama, Kazuhiko Segawa, Makiko Endo, Meganne E. Leach, Nora Brody, Brittney DeWolf, Allyn Toles, Stanley T. Fricke, Hansel J. Otero, Ulrike Grieben, Juliana Prugel, Elke Maron, Linda Pax Lowes, Volker Straub, Jordi Diaz-Manera

**Affiliations:** 1 The John Walton Muscular Dystrophy Research Centre, Translational and Clinical Research Institute, Newcastle University and Newcastle Hospitals NHS Foundation Trust, Central Parkway, Newcastle Upon Tyne, UK; 2 The Jain Foundation, Seattle, WA USA; 3 Newcastle Magnetic Resonance Centre, Translational and Clinical Research Institute, Newcastle University, Newcastle Upon Tyne, UK; 4 NMR Laboratory, Neuromuscular Investigation Centre, Institute of Myology, Paris, France; 5 St Luc University Hospital, Erasme University Hospital, Brussels and University of Liege, Liege, Belgium; 6 Center for Health Outcomes Research and Delivery Science, Division of Biostatistics and Study Methodology, Children’s National Health System, Washington, DC USA; 7 Pediatrics, School of Medicine and the Health Sciences, George Washington University, Washington, DC USA; 8 Charite Muscle Research Unit, Experimental and Clinical Research Center, A Joint Cooperation of the Charité Medical Faculty and the Max Delbrück Center for Molecular Medicine, Berlin, Germany; 9 Department of Neurology and Neurological Sciences, Stanford University School of Medicine, Stanford, CA USA; 10 The Sydney Children’s Hospital Network, and The University of Sydney, Child and Adolescent Health, Sydney, Australia; 11 Center for Gene Therapy, Nationwide Children’s Hospital, Columbus, OH USA; 12 National Institutes of Health (NINDS), Bethesda, MD USA; 13 Department of Neurology, Washington University School of Medicine, St. Louis, MO USA; 14 Friedrich Baur Institute at the Department of Neurology, University Hospital, LMU Munich, Munich, Germany; 15 Neuromuscular Unit, Department of Neurology, Hospital U. Virgen del Rocío/Instituto de Biomedicina de Sevilla, Seville, Spain; 16 APHP, Reference Center for Neuromuscular Diseases, Pitié-Salpêtrière Hospital, Institute of Myology, Paris, France; 17 Department of Neurology, National Center Hospital, National Center of Neurology and Psychiatry Tokyo, Tokyo, Japan; 18 Neuroscience Institute, Carolinas Neuromuscular/ALS-MDA Center, Carolinas HealthCare System, Charlotte, NC USA; 19 Department of Neuroscience, University of Padova, Padua, Italy; 20 The Abigail Wexner Research Institute at Nationwide Children’s Hospital, Columbus, OH USA; 21 Centro de Investigación Biomédica en Red en Enfermedades Raras (CIBERER), Barcelona, Spain; 22 Neuromuscular Disorders Unit, Neurology Department, Hospital de la Santa Creu i Sant Pau, Barcelona, Spain

**Keywords:** Limb-girdle muscular dystrophies, Dysferlin, Magnetic resonance imaging, Disease progression, Prognosis, Biomarkers

## Abstract

**Background:**

Limb-girdle muscular dystrophy R2-dysferlin related (LGMD-R2) is a progressive muscle condition with marked variability in disease course, making prognosis challenging. Quantitative MRI (qMRI) has emerged as a complementary tool that may detect progression earlier and more precisely. Integrating different data modalities is challenging with conventional approaches, and artificial intelligence (AI) can help overcome this. Our aim is to develop robust models capable of predicting clinical progression in LGMD-R2 by incorporating AI-based techniques into the analysis pipeline.

**Methods:**

Data from 188 COS 1 participants were analysed. Disease progression was assessed using the North Star Assessment for Limb Girdle type Muscular Dystrophies (NSAD). Ambulatory individuals with a maximum NSAD ≥ 20 were included, and progression trajectories were identified through hierarchical clustering. Feature selection was performed using a machine learning pipeline, and top predictors were entered into stepwise logistic regression to build clinical-only and combined clinical-MRI models.

**Results:**

Two stages of progression were identified, a fast one with a mean three-year loss of 14.4 NSAD points, and a moderate one, with a mean loss of 3.8 NSAD points. The combined model achieved better balanced accuracy than the clinical-only one (83.7% vs 78.7%). Key predictors in the combined model were disease duration and fat content measures in the anterior thigh and gracilis muscle, while the clinical model included disease duration, creatine phosphokinase (CK), and 10 m walk/run test velocity.

**Conclusions:**

Progression in LGMD-R2 can be grouped into distinct clinical trajectories. Individuals at a faster stage of progression were younger, had shorter disease duration, higher CK, greater weakness, and relatively preserved vastus intermedius and gracilis muscles. AI enabled efficient integration of heterogeneous data, and qMRI biomarkers provided complementary information that improved predictive accuracy.

**Supplementary Information:**

The online version contains supplementary material available at 10.1007/s00415-026-13868-0.

## Introduction

Limb-girdle muscular dystrophy R2 (LGMD-R2) is produced by pathogenic variants in the *DYSF* gene. This gene encodes the dysferlin protein, an integral membrane protein whose main roles are sarcolemma repair after muscle fibre injury, T-tubule formation and calcium handling [10, 12, 19]. Individuals affected usually start showing signs and symptoms of the disease in their late teens or early in their adulthood, which are characterized by muscle weakness in the lower limbs [11] and marked elevation of serum creatine phosphokinase (CK) levels [33]. This is a slowly progressive disorder with the steepest functional decline occurring during the first ten years after symptom onset [15]. Muscle weakness becomes widespread with disease progression involving the proximal and distal muscles of the upper limbs and occasionally axial muscles [3]. Along with the progression of muscle wasting, there is a reduction in CK levels due to muscle loss [11]. Respiratory muscles might show involvement in advanced stages, but no clear association with cardiomyopathy has been reported [24].

The natural history of this condition has been extensively investigated in several studies reporting considerable variability in the age at onset, rate of progression, and overall disease severity [11, 25, 29, 36]. The Clinical Outcome Study for dysferlinopathy (COS) [11] is a natural history study funded by the Jain Foundation that followed people with LGMD-R2 for up to ten years collecting different types of data. This large natural history study was made up of two shorter studies, the COS 1, running from 2012 to 2018, and the COS 2, running from 2018 to 2024, with the main aim of COS 2 being to build upon and validate the data from COS 1. Even though the median age at symptom onset was 19 years, it ranged from 3 to 60 years. Moreover, although individuals with a longer disease history often exhibited more advanced symptoms, 10% of those with less than ten years of disease history were severely affected, whereas 10% of individuals who had experienced symptoms for over 35 years were still functional and were considered to have a mild phenotype. While the cause of this variability is not yet understood, there are some factors that have been linked to worse outcome, such as earlier age at onset [15], longer disease duration [15], practising intense physical activity during teenage years [26, 28], and higher water T_2_ values in skeletal muscle MRI [23]. However, no clear genotype–phenotype correlations have been identified to date [14, 18]. Moreover, other factors such as comorbidities, medication, lifestyle, mental health and stress, nutrition, and diet, among others, which are difficult to measure in the clinical and trial setting, could also influence disease progression.

Disease progression in LGMD-R2 has traditionally been measured using clinically based outcome measures. In a one-year interim analysis of COS 1 data [27], it was concluded that, although some clinical outcome measures were sensitive to changes over six months and a year, their sensitivity was small to moderate. This led to the development and validation of a dysferlinopathy-specific motor function scale, which measured the impact of disease on daily functions, the North Star Assessment for limb-girdle type muscular dystrophies (NSAD), sensitive to change over one year [15].

Standardized response mean (SRM), a measure of sensitivity to change that is calculated by dividing the mean change by the standard deviation of that change, was used to measure this. Traditional thresholds have been established at SRM < 0.5 (small), < 0.8 (moderate), and ≥ 0.8 (large). For the clinical outcome measures evaluated during COS, all of them reached an SRM below 0.7. However, when analysing individual muscles fat fraction (FF) values in COS, not only were they sensitive to change over one and three-year periods, but also the SRMs were above 0.7 for both thigh and lower leg [35] muscles in ambulant individuals. Moreover, evidence suggests that changes in FF in whole lower limb segments (thigh and lower leg) could be as sensitive as changes in FF in individual muscles to efficiently measure progression [34]. These results build upon the growing use of quantitative MRI (qMRI) measurements as robust monitoring biomarkers.

Even though COS has been pivotal in our understanding of the natural progression of LGMD-R2, the integration and analysis of this complex dataset, including clinical, genetic and imaging variables, is still a major limitation. Most publications stemming from large databases are unidimensional, focussing on specific data modalities at a time rather than trying to integrate the information. This fragmented approach might overlook meaningful patterns that could emerge from a more comprehensive analysis. Here, we aimed to integrate the different types of data collected in COS 1 to develop robust models useful to predict the progression of individuals with LGMD-R2. In this context, the inclusion of artificial intelligence (AI)-based techniques in the pipeline analysis could offer a powerful tool to explore multidimensional interactions and identify novel insights into disease progression and heterogeneity [6].

AI has proven to be particularly useful for handling this type of complex datasets that include variables with different natures, especially when they are acquired longitudinally. Traditional statistical methods usually rely on predefined assumptions and can only handle a limited number of variables at a time; however, AI models can process high-dimensional data and recognize hidden patterns that might not be apparent through standard statistical analysis [9, 32]. In a systematic methodological review focussing on the use of AI for the analysis of longitudinal data [5], it was shown how an AI-based approach trained on longitudinal electronic health records allowed for the prediction of outcomes, such as disease progression and response to treatment, through the integration of changes over time in clinical, demographic, and biochemical variables. Applying similar approaches to LGMD-R2 could potentially help stratify individuals, identify biomarkers of disease progression, and better understand the role of different factors in shaping disease severity.

## Materials and methods

### Study design and participants

The COS 1 study prospectively collected demographic, genetic, clinical, and imaging data of 188 individuals for up to five years. Eligible participants had two confirmed pathogenic variants in the *DYSF* gene or one pathogenic variant plus absent dysferlin expression measured through immunoblot in muscle tissue and/or monocytes. A detailed description on the methods of this natural history study is available in the initial report from the COS 1 study [11].

### Variable selection

The COS 1 database contained 307 individual variables of diverse nature collected at different time-points (Supplementary Table S1). Some variables were initially excluded from this analysis due to the following reasons: the Performance of the Upper limb scale (PUL) [21] and lying forced vital capacity (FVC) had only been measured after year 1; therefore, the changes over time were partial and missing for the first visits; in a previous paper [23] from COS 1, when analysing qMRI data, only the water T_2_ values from the Paris and Newcastle sites had been used to limit the impact of inter-site variability, and when included in our preliminary analysis, results were discordant and noisy; genetic variants were also removed because, due to the multiplicity of options, very few of them could be grouped to achieve sufficient representation; only the total score for the NSAD was used, removing the 29 individual items to avoid collinearity; and the Egen Klassifikation (EK) score was only calculated for non-ambulant individuals, and as all included participants were ambulant, this was removed. Previous research showed that muscle FF values were equal between both sides of the body [2], so the average left–right value was used. A preliminary analysis showed that the same applied to manual muscle testing (MMT) measurements (Supplementary Table S2), except for elbow flexion (biceps and brachioradialis) and shoulder flexion. Therefore, the maximum value for each movement for each individual at each visit was retained, consistent with the approach already applied to the hand-held dynamometer measurements. In line with the current evidence suggesting that the analysis of a segment’s FF could be as good as the analysis of individual muscles’ FF to describe disease progression [34], which would simplify qMRI segmentation and FF analysis, muscles were grouped by localization into thigh anterior compartment (vastus medialis, vastus intermedius, and vastus lateralis), thigh posterior compartment (biceps femoris, semitendinosus, and semimembranosus), thigh medial compartment (adductor magnus, gracilis, and sartorius), lower leg anterior compartment (tibialis anterior and extensor digitorum muscles), and lower leg postero-lateral compartment (peroneus muscles, gastrocnemius medialis, gastrocnemius lateralis, and soleus).

### Trajectory modelling

To predict clinical progression, the NSAD was selected as the target variable. The NSAD is a validated functional scale developed to capture disease progression in people with LGMD and reflect clinically meaningful changes over time [15, 16]. The score goes from zero to 54, with higher values reflecting better motor performance. Patients are asked to perform 29 tasks, each scored with a maximum of one or two points depending on the item. Most items assess lower limb function (walking, running, jumping, hoping, climbing and descending stairs, among others), while four out of 29 focus on axial and upper limb function. As a result, the NSAD better reflects lower limb function rather than trunk or upper limb. The NSAD is an ordinal scale [15, 16]; consequently, measuring change in a consistent and comparable way across all disease stages is complex. So, to ensure that the measured changes were as clinically comparable as possible, only ambulant individuals at baseline with a maximum NSAD score equal or above 20 were included.

Trajectories of NSAD progression were defined in an unsupervised way, as presented by Bhagwat et al. [1]. Patients with non-missing consecutive visits were selected for the modelling. A higher number of visits (N) enable the modelling of NSAD over a longer period; however, patient drop-off reduces the size of the modelling set for larger N values. The largest drop was observed between the fifth and sixth visits (year 3 and year 4 visits, respectively), declining from 73 to 26 patients (Supplementary Fig. S1). Based on this analysis, we established a minimum threshold of five complete visits for patient inclusion in the modelling cohort.

Hierarchical clustering was applied on the modelling cohort, using Euclidean distances and the Ward method. Clustering on the absolute NSAD scores led to clusters representing the overall disease stage rather than different trajectories; therefore, the NSAD scores were transformed by subtracting the maximum NSAD score of each patient to each one of their visits, representing absolute change in this scale rather than absolute value.

For each selected cluster, the average trajectory template was calculated. When assigning a trajectory to the complete cohort, the Euclidean distance (ignoring missing time-points) between each patient and each template was calculated, and the cluster with the lowest distance was assigned.

### Feature selection

Collinearity and information redundancy across features were tackled by incorporating a feature selection step. This consisted of a Machine Learning (ML)-based model tasked with predicting the progression type of the patients given a single timepoint data. The pipeline starts with a Recursive Feature Optimization process to select the 30 most discriminating features, used to train a final Random Forest classifier. The pipeline was trained and evaluated using stratified cross-validation, grouping all patient time-points together to avoid data leakage. The SHapley Additive exPlanations (SHAP) framework for AI explainability was used to obtain each feature’s importance, which were used to manually choose the most relevant variables [20].

First, based on the AI explainability results, the variables with the highest mean SHAP values were tested for significant differences between the two clusters using the non-parametric Mann–Whitney test with Benjamini–Hochberg correction for multiple comparisons. Second, a forward stepwise (likelihood ratio) variable selection procedure was used to build a binary logistic regression model, identifying predictors of disease progression. The predicted probabilities derived from this analysis were used to build a receiving operator curve (ROC) to evaluate the discriminatory ability of the model. Two models were built, one including only clinical data and one including clinical and qMRI data.

The target definition and feature selection were performed on Python 3.12.

## Results

**Fig. 1:**
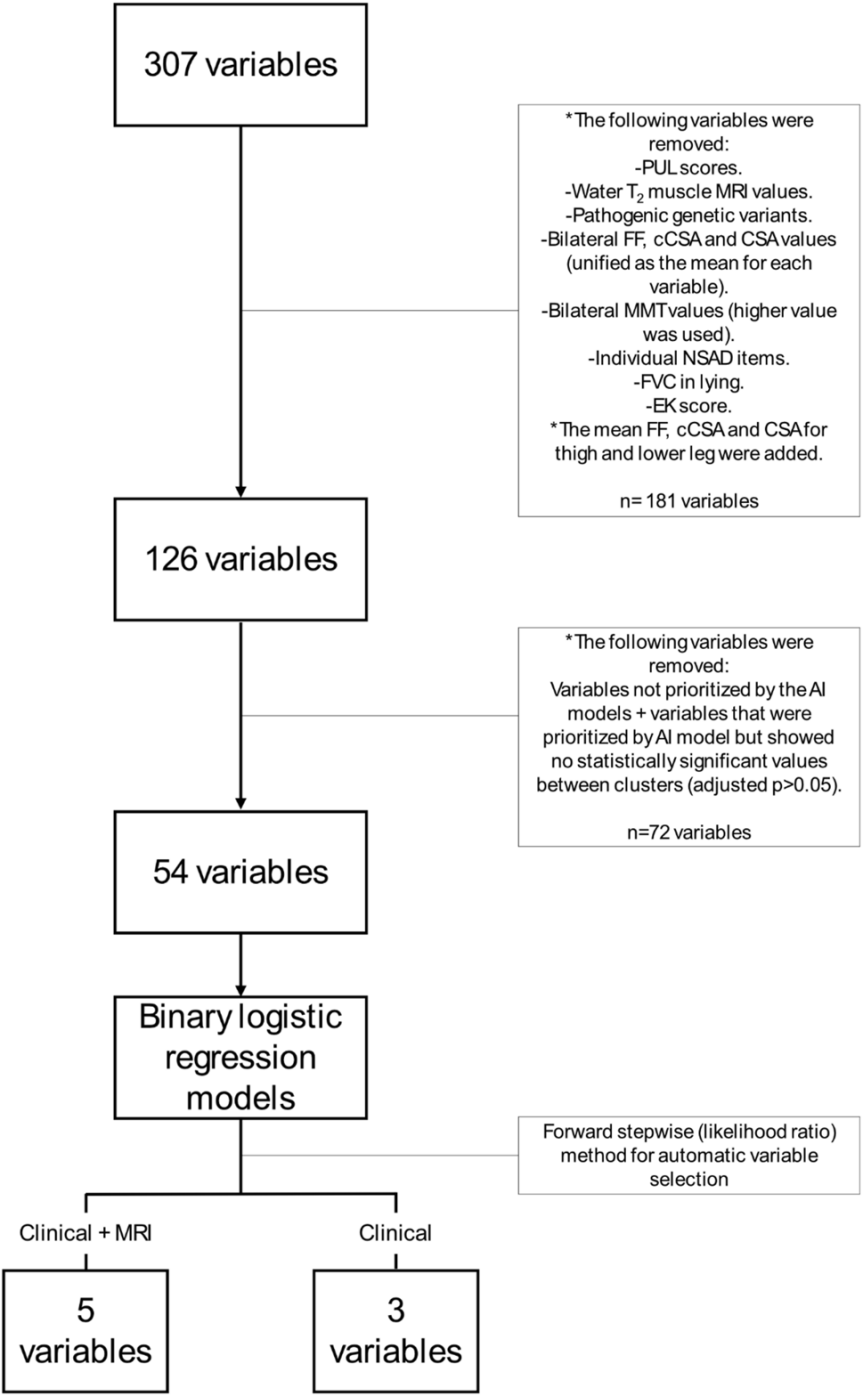
Workflow for variable selection. *AI* artificial intelligence, *cCSA* contractile cross-sectional area, *CSA* cross-sectional area, *EK* Egen Klassifikation, *FF* fat fraction, *FVC* forced vital capacity, *MMT* manual muscle test, *MRI* magnetic resonance imaging, *NSAD* North Star Assessment for limb-girdle type muscular dystrophy, *PUL* performance of the upper limbs

**Fig. 2:**
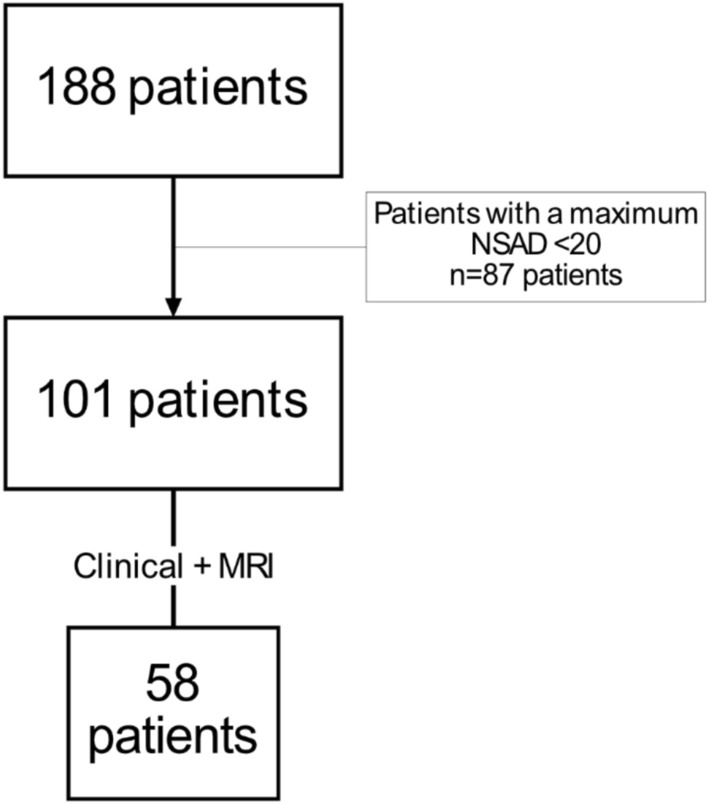
Workflow for patient selection. *MRI* magnetic resonance imaging, *NSAD* North Star Assessment for limb-girdle type muscular dystrophy

The original database contained information of 307 variables from 188 individuals followed up for an average of 1280.8 days (R 182–1909 days) (approximately 3 years and 6 months). However, following the modifications to the database described in the methods section (patient selection, exclusion of certain variables, simplification of variables with bilateral data, and muscle grouping by function and localization), the initial working database comprised 101 patients (58 of them had at least one qMRI scan) (Fig. 1) and 126 individual variables (Fig. 2).

This cohort was made up of 53 women (52.5%), most of them with a White/Caucasian ethnical background (71.3%) followed by those with an Asian background (15.8%). In immunoblotting, dysferlin was absent in 82.9% of patients and reduced in the remaining. At baseline, the mean age at assessment was 35.2 years (y) (± 12.6 y), age at symptom onset was 23.1 y (± 10.2 y), and the mean disease duration was 12.1 y (± 7.7 y). The mean NSAD score was 35 (± 12), and on average, patients were slightly overweight (BMI 25.4 ± 5.8).

**Fig. 3:**
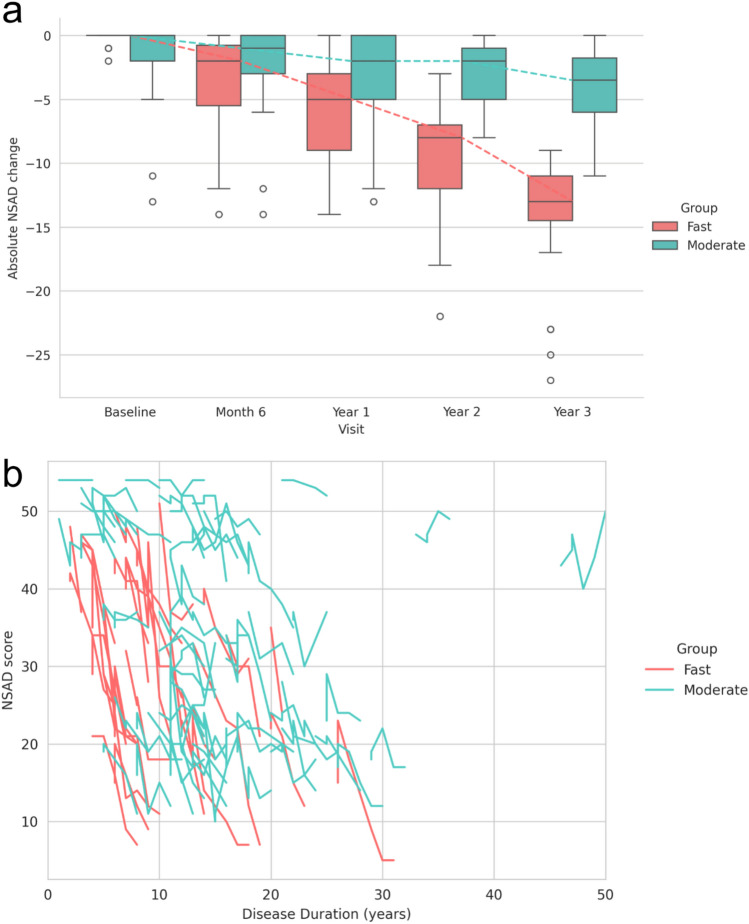
**A** Boxplot showing NSAD trajectory for each identified stage of progression over a three-year period. Figure 3b Individual patients’ NSAD trajectories coloured by stage of progression based on disease duration. NSAD: North Star Assessment for limb-girdle type muscular dystrophy

Two characteristic NSAD stages of progression were identified: a fast stage of progression (Fa) (*n* = 30) and a moderate stage of progression (Mo) (*n* = 71). Patients in the fast stage showed an absolute decrease in NSAD of 14.4 points (± 4.7) over three years, while the ones in the moderate stage showed a decline of 3.8 (± 2.8) NSAD points during the same period (Fig. 3a, b).

AI explainability techniques (SHAP analysis) were performed to understand which variables the model prioritized to predict patients’ trajectories. The highest mean SHAP values are found in Supplementary Table S3.

A total of 54 variables were significantly different between clusters (Supplementary Table S4). Patients in the fast stage of progression were younger at assessment (Fa 29 y vs Mo 35 y, *p* < 0.001), had a shorter disease duration (Fa 8 y vs Mo 14 y, *p* < 0.001), and higher CK levels (Fa 6630 U/L vs Mo 3486 U/L, *p* < 0.001). They were overall weaker measured through both MMT (for example, hip extension Fa 3.8 vs Mo 6.0, *p* < 0.001) and hand-held dynamometer (for example, grip Fa 81.0 lb vs Mo 90.0 lb, *p* < 0.01), and performed slower in multiple timed tests (for example, time to rise from floor Fa 5.7 s vs Mo 4.6 s, *p* < 0.01). Even though clinically they were weaker, from an qMRI point of view there were some specific muscles that were less affected in those individuals going through a fast stage progression: gracilis (Fa 7.9% vs Mo 11.3%, *p* = 0.048), sartorius (Fa 10.8% vs Mo 13.5%, *p* = 0.044), vastus intermedius (Fa 17.8% vs Mo 39.5%, *p* < 0.001), and vastus medialis (Fa 22.3% vs Mo 38.2%, *p* = 0.036).

**Fig. 4:**
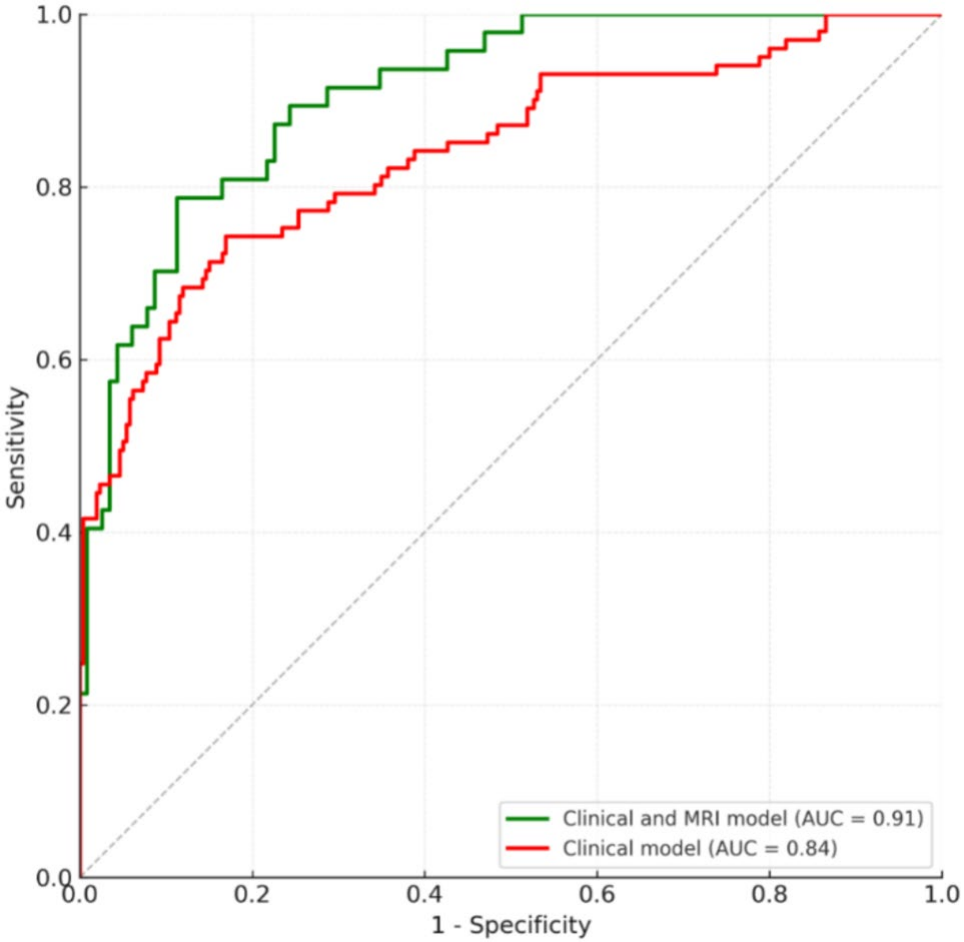
Receiving operator curve for the clinical-only model (red) and the clinical and MRI model (green). *AUC* area under the curve, *MRI* magnetic resonance imaging

Two binary logistic regression models were built and their corresponding ROC curves: one including clinical and imaging outcomes, and one including only clinical variables. The model that included both data types achieved the highest performance. It was able to explain 59% (Nagelkerke *R*^2^: 0.587) of the variability and was able to differentiate between groups better than chance (X^2^ (5) = 85.77, *p* < 0.001). Shorter disease duration, a relatively preserved vastus intermedius compared to the vastus medialis and lateralis, and higher contractile muscle tissue in the gracilis were linked to a faster decline over the next three years (Table 1). Using the predicted values, the ROC analysis indicated an area under the curve (AUC) of 0.91 (95% CI: 0.86–0.95). The optimal cutoff point was 0.366 (predicted probabilities equal or greater than 0.366 were classified as positive), with a balanced accuracy of 83.7%, sensitivity of 78.7%, and a specificity of 88.7% (Fig. 4).

**Table 1:** Clinical and MRI binary logistic regression model (table and equation)

Predictor	*B (β)*	SE	Wald *χ*^2^	*p*	OR	95% CI OR
FF VI	− 0.516	0.113	20.7	< 0.001	0.60	0.48–0.74
cCSA G	0.007	0.002	9.9	0.002	1.01	1.01–1.02
FF ant thigh	0.499	0.114	19.2	< 0.001	1.65	1.32–2.06
cCSA ant thigh	− 0.003	0.001	8.6	0.003	0.99	0.99–0.99
Disease duration	− 0.324	0.072	20.1	< 0.001	0.72	0.63–0.83
Constant	3.697	1.402	6.9	0.008	40.33	

$$p=\frac{1}{1+{e}^{-(3.697-0.324\times dd+0.499\times FFat-0.516\times FFVI+0.007\times cCSAG-0.003\times cCSAat)}}$$

*Ant* anterior, *at* anterior thigh, *cCSA* contractile cross-sectional area, *CI* confidence interval, *dd* disease duration, *FF* fat fraction, *G* gracilis, *OR* odds ratio, *SE* standard error, *VI* vastus intermedius

Given that MRI, and particularly the qMRI required to calculate FF, is not available in all neuromuscular centres worldwide, an additional model relying exclusively on clinical variables was built. This model explained 44% (Nagelkerke *R*^2^: 0.443) of the variability and was also significantly better than chance to classify patients (*X*^2^ (3) = 132.86, *p* < 0.001). According to this model, patients at risk of progressing faster over the next three years were those with shorter time since symptom onset, higher CK levels in blood, and those slower when walking or running 10 m (10MWTV) (Table 2). The ROC analysis indicated an AUC of 0.84 (CI 95% 0.79–0.89) with an optimal cutoff point at 0.317 (predicted probabilities equal or greater than 0.317 were classified as positive) achieving a balanced accuracy of 78.7%, sensitivity of 74.3%, and a specificity of 83.1% (Fig. 4).

**Table 2:** Clinical binary logistic regression model (table and equation)

Predictor	*B (β)*	SE	Wald *χ*^2^	*p*	OR	95% CI OR
CK (1000 U/L)	0.353	0.057	39.0	< 0.001	1.42	1.28–1.59
Disease duration	− 0.133	0.031	18.9	< 0.001	0.88	0.82–0.93
10MWR velocity	− 1.730	0.284	37.0	< 0.001	0.18	0.10–0.31
Constant	1.009	0.641	2.5	0.115	2.74	

$$p=\frac{1}{1+{e}^{-(1.009-0.133\times dd+0.353\times CK-1.73\times 10MWRV)}}$$

*10MWR* 10 m walk/run test, *CI* confidence interval, *CK* creatine phosphokinase, *OR* odds ratio, *SE* standard error, *V* velocity

There was a 77.9% inter-model agreement in the predicted outcomes. Among these concordant predictions, 71.0% were correct and 6.9% were incorrect. In the cases where the models disagreed, the combined model made the correct prediction 15.2% of the times, whereas the clinical model was correct 6.9%.

To facilitate clinical implementation (bearing in mind this is a research project and the limitations that are discussed below), we developed a web-based interface in which users can input the values of the variables for each model (http://www.myoguide.org/tools/dysf-stage-progression-prediction/). The platform automatically applies the model equation (Tables 1, 2), calculates the predicted probability, and compares this value against the optimal ROC-derived cutoff. Based on this, the tool classifies each individual as going through a fast or moderate stage of progression.

## Discussion

This study reflects how the incorporation of AI-based analytical techniques can enable a deeper and more systematic analysis of large and complex datasets. In our case, these methods allowed the grouping of individuals according to their clinical trajectories in an unsupervised manner without manual input and handling a large number of variables and sequentially exclude those that were not relevant to our primary objective of predicting disease progression. Ultimately, it contributed to the development of two predictive models, one based on clinical data and the other combining clinical and qMRI data, that accurately classified individuals according to their clinical evolution into one of two stages of progression over a three-year period, with the combined model outperforming the clinical one.

Our findings are consistent with the growing evidence that muscle imaging, qMRI in particular, is a powerful tool to study neuromuscular diseases, a promising multimodal biomarker, and that it should be included along with functional and timed tests in future clinical trials [7, 13, 22, 30]. The combined model outperformed the clinical one, achieving a higher balanced accuracy, sensitivity, and specificity, showing superior discriminatory capacity on the ROC analysis, despite more individuals and datapoints being included in the clinical model because only half of the patients had a qMRI scan. Moreover, it was able to explain more data variance than the clinical variables on their own, reflecting that qMRI intrinsically carries information that is not captured by the functional tests.

Individuals going through a fast stage of progression were younger and in earlier stages of their disease, as had previously been reported [15]. Interestingly, disease duration was the only time-related variable retained in the models, emphasizing its importance to understand disease stage in individuals. Although recall bias might impact the precision of this measure, our findings suggest that independently of baseline clinical severity, the time since patient-reported symptom onset is a key determinant of the rate of progression over the following years. Moreover, these individuals had higher CK levels, were clinically weaker based on MMT and HHD, and performed slightly worse in the timed tests; however, when looking at the qMRI, some muscles from the anterior and medial thigh were less replaced by fat, showing higher content of contractile muscle tissue. The gracilis and vastus intermedius in particular were the ones retained in the combined model, and when sparred, these findings increased the odds of a patient going through a fast stage of progression, especially if there was a discrepancy between the vastus intermedius and the medialis and lateralis, with these latter two relatively more replaced by fat. This heterogeneous involvement of the anterior thigh muscles is also evidenced through the quadriceps “diamond” sign, a clinical finding frequently described in people affected by LGMD-R2, in which patients show an asymmetric bulge in their anterior thigh when asked to stand with their knees flexed [8, 31]. Additionally, quadriceps muscles are essential for stabilizing the knee when patients stand, walk, or climb up or down stairs. Based on our clinical experience, individuals with weaker quadriceps also score lower in the NSAD and other clinical tests. Therefore, it is reasonable that those individuals who still have considerable remaining contractile muscle tissue in the quadriceps muscles measured by qMRI, but whose muscles are not completely intact, will have a higher chance of progressing at a faster rate over a short period of time. In fact, this statement is reinforced by our own results exploring water T_2_ as a potential predictor of disease progression. In that study, we identified that higher water T_2_ values in the vasti muscles, indicative of an active muscle degeneration process, were associated with worse prognosis [23].

CK is an enzyme found in several domains of the muscle fibre, and is therefore an unspecific marker of muscle damage [4]. In LGMD-R2, values are elevated early in the disease course, and they progressively fall as individuals age and the disease progresses, likely reflecting the decline in overall contractile muscle mass [11]. The inclusion of the CK in the clinical model suggests that, despite it not reliably correlating to disease severity, it carries unique information on an individual’s disease stage. This is consistent with previous analyses [35] which identified associations between CK levels, disease duration, FF, water T2 times, and baseline phosphorus MR spectroscopy (PMRS) values. Together, these findings support the idea that CK captures aspects of the condition that neither disease duration nor mobility can fully explain, improving the accuracy of the model in predicting stage of progression over the coming three years.

The clinical variables identified by the model, CK levels, 10MWTV, and disease duration, are consistent with established clinical knowledge and capture fundamental dimensions of disease severity and progression in dysferlinopathy, with its identification as relevant variables reinforcing existing knowledge. Moreover, our study highlights their combined relevance specifically in predicting disease progression, an area where longitudinal evidence remains comparatively limited. Furthermore, in the context of neuromuscular disorders, where numerous outcome measures can be collected but time and resources in both clinical practice and trial settings are constrained, the identification of a small set of readily available variables with meaningful predictive value may help simplify assessments. This could allow clinicians and researchers to focus on the most informative measures, reducing burden while still capturing key aspects of disease evolution.

These findings are relevant as they could improve inclusion criteria and stratification in future clinical trials, given that most interventional studies last between one and three years, with a maximum of around five if there is a long-term extension phase. Accounting for this could help minimize the confounding effect disease stage and individual disease progression rate could have on an intervention’s efficacy. Moreover, even though this condition has traditionally been described in the literature as a “late onset, slowly progressive” disorder, the LGMD patient community does not fully agree with this, highlighting its progressive and irreversible nature and how time is not on their side, voicing concerns about the length of clinical trials [37].

Individuals were clustered based on three-year follow-up data; therefore, our results cannot be extrapolated to longer periods of time. Repeating the analysis on a larger dataset with more individuals and longer follow-up periods would allow us to improve our understanding on disease natural history. It could help determine how long this actively declining phase lasts before individuals plateau, whether this is consistent among individuals or some have shorter or longer declines, if all patients deteriorate at the same rate, and if this can be predicted.

Another limitation is the fact that by including disease duration in the models, individuals who do not show any signs or symptoms cannot be reliably assessed through them. If we were to evaluate an asymptomatic individual, the model would technically allow us to input a disease duration of zero, which should return a higher probability, and therefore classify the individual as being at a fast stage of progression. However, this would not fully reflect the natural history of this condition. An asymptomatic 16-year-old and an asymptomatic 30-year-old (this latter situation being rare but seen in our clinical experience) would not be expected to have the same chances of entering a fast stage of progression over the following years. The fact that an individual has remained asymptomatic past their twenties already suggests a more indolent lifelong course, and it is less likely that they will ever go through the fast progression stage previously described in our cohort. Although the model incorporates other variables, qMRI measurements are expected to be similar between asymptomatic individuals as they correlate with muscle strength, and CK could potentially be higher in younger patients. However, because age at assessment is not included, the model may underestimate these differences and oversimplify the predicted trajectory for these cases.

Moreover, we only included ambulant individuals as we based our definition of progression on the NSAD scale. This is not a minor limitation of our study, as it restricts the generalisability and clinical applicability of our findings. A proportion of individuals with dysferlinopathy will eventually lose ambulation and are therefore excluded from our analysis. However, this decision was made deliberately, as including non-ambulant individuals would have introduced substantial heterogeneity. In such cases, a lack of measurable progression on the NSAD may not reflect true disease stability, but rather a floor effect, where individuals have limited remaining function to lose. Consequently, grouping early-stage, slowly progressing individuals with those who are non-ambulant would not be physiopathologically comparable. Furthermore, given that clinical trials in this field often prioritize ambulant individuals in earlier disease stages, we chose to align our approach with this context. This issue also reflects the existing need for an outcome measure able to linearly project patients at all severity stages. If individuals were clustered using a scale that looked into upper limb function which is involved later in the disease, like the PUL scale [17], this would help provide further insight into the severity and impact of upper limb weakness, whether the progression is slow over time or whether there is a period of more marked decline like the one described in the lower limb function. Unfortunately, PUL data were not available from baseline in COS 1, limiting our ability to explore this aspect. Future analyses using COS 2 data, where this scale has been consistently collected from baseline, will help address this gap.

Additionally, our study showed that the different data modalities and features collected often express collinearity and information redundancy. Our AI-based strategy for feature selection prioritized quantitative measures over tests relying on patients’ cooperation, suggesting they are able to better capture the latent disease information with minimal variance. This finding is critical to the design of prospective studies, helping minimize data collection efforts while ensuring information richness. At the same time, the complexity of the database and the high degree of correlation and information overlap between variables constitute a significant limitation, as they reduce the ability to draw robust and unbiased conclusions.

Last but not least, our conclusions should be validated with an independent cohort of individuals with LGMD-R2 to increase their robustness and generalisability, and to confirm that the performance of the proposed models is maintained across different populations. External validation is a key step to ensure that the observed associations and predictive metrics are reproducible and not specific to the current dataset. While our findings provide a strong basis for further investigation, such validation will be essential before these approaches can be confidently applied in clinical practice or trial settings.

In conclusion, our findings emphasize how the clinical rate of progression that an individual is currently experiencing varies according to disease stage and stress the value of integrating multiple data modalities to maximize the knowledge derived from the analysis, in this case by integrating AI-based techniques into the methods. By identifying a cluster of individuals at a higher risk of a faster functional decline, we present a model that could refine inclusion criteria, stratification, and trial design. On the one hand, enrolling patients going through a fast progression phase could increase the likelihood of detecting a treatment effect in a shorter period, as measurable change is occurring faster. On the other hand, individuals in a phase of rapid decline may have entered a cascade of pathological events that is less amenable to therapeutic modification, potentially limiting the observable benefit of an intervention. Nevertheless, accurately identifying the disease progression stage is essential for appropriate interpretation of treatment effects and clinical outcomes. The fact that the combined model outperformed the clinical-only one highlights the importance of qMRI as a biomarker, and its inclusion in natural history studies and interventional clinical trials alongside clinical outcome measures. Future work on larger and independent cohorts is essential to validate these observations. Moreover, an extended follow-up and the analysis of progression in non-ambulant individuals using scales measuring upper limb performance could lead to a more in-depth phenotyping of the more severely affected individuals with LGMD-R2 and ultimately assessing whether the expected change is substantial enough to allow a treatment effect to be reliably detected, and therefore, this group of individuals be included in interventional trials.

## Supplementary Information

Below is the link to the electronic supplementary material.

Supplementary file1 (PDF 277 KB)
