# Supplementary material for: Identification of prognostic biomarkers in a large cohort of patients with LGMD R2
Source: J Neurol. 2026 May 26;273(6):344. doi: 10.1007/s00415-026-13868-0 (PMC13212672; doi:10.1007/s00415-026-13868-0)
Supplement: Supplementary file 1 — Supplementary file1 (PDF 277 KB) [file 415_2026_13868_MOESM1_ESM.pdf]

Supplementary material

to

Identification of prognostic biomarkers in a large cohort of patients with LGMD R2

Carla F. Bolano-Diaz[1*], Jose Verdu-Diaz[1*], Dan Hao[1], Meredith K. James[1], Laura E. Rufibach[2], Andrew M. Blamire[3], Harmen Reyngoudt[4], Pierre G. Carlier[4,5], Heather Gordish-Dressman[6,7], Heather Hilsden[1], Simone Spuler[8], John W. Day[9], Kristi J. Jones[10], Diana X. Bharucha-Goebel[11,12], Alan Pestronk[13], Maggie C. Walter[14], Carmen Paradas[15,16], Tanya Stojkovic[17], Madoka Mori-Yoshimura [†18], Elena Bravver[†19], Elena Pegoraro[20], Jerry R. Mendell[21], The Jain COS Consortium[2], Volker Straub[1**], Jordi Diaz-Manera[1,22,23**]

1. The John Walton Muscular Dystrophy Research Centre, Translational and Clinical Research Institute, Newcastle University and Newcastle Hospitals NHS Foundation Trust, Central Parkway, Newcastle Upon Tyne, United Kingdom
2. The Jain Foundation, Seattle, Washington, USA
3. Newcastle Magnetic Resonance Centre, Translational and Clinical Research Institute, Newcastle University, United Kingdom
4. NMR Laboratory, Neuromuscular Investigation Centre, Institute of Myology, Paris, France
5. St Luc University Hospital, Erasme University Hospital, Brussels and University of Liege, Belgium.
6. Center for Translational Science, Division of Biostatistics and Study Methodology, Children's National Health System, Washington, DC, USA
7. Pediatrics, Epidemiology and Biostatistics, George Washington University, Washington, DC, USA
8. Charite Muscle Research Unit, Experimental and Clinical Research Center, a joint cooperation of the Charité Medical Faculty and the Max Delbrück Center for Molecular Medicine, Berlin, Germany
9. Department of Neurology and Neurological Sciences, Stanford University School of Medicine; Stanford, CA, USA
10. The Children's Hospital at Westmead, and The University of Sydney, Australia
11. Center for Gene Therapy, Nationwide Children's Hospital, Columbus, OH, USA
12. National Institutes of Health (NINDS), Bethesda, MD, USA
13. Department of Neurology Washington University School of Medicine, St. Louis, MO, USA
14. Friedrich Baur Institute at the Department of Neurology, University Hospital, LMU Munich
15. Neuromuscular Unit, Department of Neurology, Hospital U. Virgen del Rocío/Instituto de Biomedicina de Sevilla, Sevilla, Spain
16. Centro de Investigación Biomédica en Red en Enfermedades Neurodegenerativas (CIBERNED), Spain
17. APHP, Reference Center for Neuromuscular Diseases, Pitié-Salpêtrière Hospital, Institute of Myology, Paris, France
18. Department of Neurology, National Center Hospital, National Center of Neurology and Psychiatry Tokyo, Japan
19. Neuroscience Institute, Carolinas Neuromuscular/ALS-MDA Center, Carolinas HealthCare System, Charlotte, NC, USA
20. Department of Neuroscience, University of Padova, Italy
21. The Abigail Wexner Research Institute at Nationwide Children's Hospital, Columbus, Ohio USA
22. Centro de Investigación Biomédica en Red en Enfermedades Raras (CIBERER), Barcelona, Spain
23. Neuromuscular Disorders Unit, Neurology Department, Hospital de la Santa Creu i Sant Pau, Barcelona, Spain

*These authors contributed equally to this work and share first authorship

**These authors contributed equally to this work and share senior authorship

[†] Deceased

Corresponding author: Carla F Bolano-Diaz, carla.bolano-diaz@newcastle.ac.uk

Supplementary methods

Table S1 Data collected in COS 1.

Data	Baseline	Month 6	Month 12	Month 24	Month 36	Month 48	Month 60
Gender	X						
Ethnicity	X						
Disease duration	X	X	X	X	X	X	X
Age at symptom onset	X						
Genetic variants	X						
Residual dysferlin expression (IB Bx and Mo, IHC Bx)	X						
Exercise levels as teenagers	X						
Body mass index	X	X	X	X	X	X	X
Creatine phosphokinase	X		X	X	X	X	X
NSAD total score and 29 individual subitems (or converted from NSAA/MFM)	X	X	X	X	X	X	X
6MWT (distance and velocity)	X	X	X	X	X	X	X
10MWT (distance and velocity)	X	X	X	X	X	X	X
TC4S (time and velocity)	X	X	X	X	X	X	X
TD4S (time and velocity)	X	X	X	X	X	X	X
TRF (time and velocity)	X	X	X	X	X	X	X
TUG (time and velocity)	X	X	X	X	X	X	X
Manual muscle testing (right and left) (UL and LL)	X	X	X	X	X	X	X
Handheld myometry (max value) (UL and LL)	X	X	X	X	X	X	X
PUL test				X	X	X	X
MFM 32-p scale (Domains 1, 2 and 3, total score)	X	X	X	X			
ACTIVLIM total score	X	X	X	X	X	X	X
EK total score (NA)	X	X	X	X	X	X	X
Brooke test	X	X	X	X			
FVC sitting position (L and %)	X	X	X	X	X	X	X
FVC lying position (L and %)				X	X	X	X
MRI: FF skeletal muscle (right and left) (LL)*	X		X	X	X		
MRI: cCSA/CSA skeletal Muscle (right and left) (LL)*	X		X	X	X		
MRI: T_2 water values skeletal muscle (right and left) (LL)*	X		X	X	X		

6MWT: 6 minute walk test, 10MWT: 10 minute walk test, ACTIVLIM: Activity limitatios questionnaire, Bx: Biopsy, CSA: Cross sectional area, cCSA: Contractile cross sectional area, IB: Immunoblot, EK: Eglen Klassifikation scale, FF: Fat fraction, FVC: Forced vital capacity, IHC: Immunohistochemistry, LL: Lower limbs, MFM: Motor function measurement, Mo: Monocytes, MRI: Magnetic resonance imaging, NA: Non-ambulant, NSAA: Northstar ambulatory assessment, NSAD: Northstar assessment for limb girdle muscular dystrophy, PUL: Performance of the upper limbs, TC4S: Time to climb four steps, TD4S: Time to descend four steps, TRF: Time to rise from floor, TUG: Time up and go, UL: Upper limbs.

Table S2 Linear mixed model results comparing muscle strength measure through manual muscle testing between left and right sides, adjusted for age

Movement	Estimate of fixed effects (left side)	95% Confidence interval	p value
Ankle dorsiflexion	-0.103	-0.253 – 0.046	0.176
Ankle eversion	0.060	-0.096 – 0.216	0.451
Ankle inversion	-0.145	-0.295 – 0.005	0.059
Ankle plantarflexion (knee straight)	0.025	-0.185 – 0.235	0.816
Ankle plantarflexion (knee flexed)	0.047	-0.105 – 0.199	0.543
Elbow extension	-0.003	-0.105 – 0.100	0.960
Elbow flexion brachioradialis	0.190	0.083 – 0.297	<0.001
Elbow flexion biceps	0.143	0.038 – 0.247	0.008
Hip adduction	0.038	-0.093 – 0.169	0.565
Hip abduction	0.056	-0.095 – 0.206	0.469
Hip extension	0.047	-0.087 – 0.181	0.489
Hip flexion	0.019	-0.099 – 0.137	0.755
Knee extension	0.031	-0.090 – 0.153	0.611
Knee flexion	-0.005	-0.110 – 0.100	0.921
Shoulder abduction	-0.010	-0.127 – 0.107	0.865
Shoulder flexion	0.116	0.005 – 0.226	0.041
Wrist extension	-0.023	-0.110 – 0.065	0.611
Wrist flexion	0.062	-0.049 – 0.172	0.273

Fig. S1 Number of patients with non-missing consecutive visits

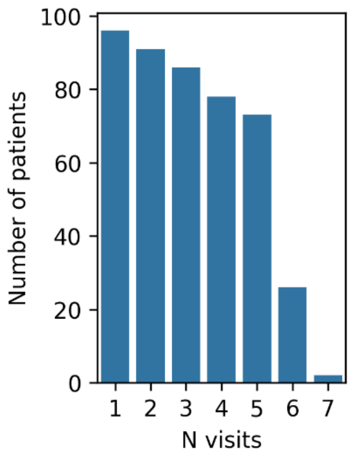

Supplementary results

Table S3 Highest mean SHAP values for purely clinical models (a) and models including MRI data (b) individual muscle data and (c) segmental muscle data

a)

Variable	Mean SHAP value
Time to climb 4 stairs - velocity	0.0609
Creatine phosphokinase	0.0519
MMT - Shoulder Abduction	0.032
MMT - Ankle Dorsiflexion	0.0318
MFM Domain 2 score	0.0256
MMT - Ankle Plantarflexion (knee straight)	0.0234
HHM - Elbow flexion brachioradialis	0.0232
Time to climb 4 stairs	0.0197
Time to run/walk 10 meters - velocity	0.0186
HHM - Hip abduction	0.0159
MMT - Hip Extension	0.012
Time to descend 4 stairs	0.0118
MMT - Ankle Plantarflexion (knee flexed)	0.0117
MMT - Hip Abduction	0.0105
HHM - Knee extension	0.0095
MFM Total score calculated with imputations	0.0089
MMT - Hip Adduction	0.0087
HHM - Hip adduction	0.0086
HHM - Pinch grip	0.0065
MFM Domain 3 score	0.0065
MMT - Elbow Extension	0.0063
HHM - Knee flexion	0.0058
MMT - Elbow Flexion Brachioradialis	0.0055
MMT - Hip Flexion	0.0053
Muscle biopsy WB	0.0052
Gender	0.0051
MMT - Ankle Inversion	0.005
Ethnicity	0.0046
HHM - Ankle dorsiflexion	0.0046
Age at first muscle symptom(s)	0.0043

b)

Variable	Mean SHAP value
Creatine phosphokinase	0.0455
FF - Semitendinosus	0.0407
Time to run/walk 10 meters	0.0287
HHM - Wrist extension	0.0226
CSA - Biceps femoris	0.0219
cCSA - Vastus lateralis	0.0207
MMT - Elbow Flexion-biceps	0.0187
MMT - Knee Flexion	0.0186
CSA - Soleus	0.0176
Time to rise from floor	0.0167
6MWT - velocity	0.0147
HHM - Pinch grip	0.0145
MFM Domain 1 score	0.0144
MFM Total score	0.0125
CSA - Gastrocnemius medialis	0.0123
Muscle biopsy IHQ	0.0123
CSA - Adductor magnus	0.0110
FF - Peroneous	0.0105
CSA - Extensor digitorum	0.0090
MMT - Shoulder Abduction	0.0084
MMT - Ankle Plantarflexion (knee straight)	0.0082
Time up and go	0.0073
MFM Domain 2 score	0.0065
CSA - Semimembranosus	0.0052
MMT - Ankle Inversion	0.0050
Age at first muscle symptom(s)	0.0045
HHM - Ankle plantar flexors (knee straight)	0.0044
MMT - Ankle Eversion	0.0043
cCSA - Semimembranosis	0.0042
MFM Domain 3 score	0.0038

c)

Variable	Mean SHAP value
Time to climb 4 stairs - velocity	0.0531
Creatine phosphokinase	0.0529
MMT - Hip Abduction	0.0281
FF - Thigh posterior compartment	0.0246
MMT - Elbow Flexion Brachioradialis	0.0243
MMT - Wrist Extension	0.0202
CSA - Lower leg anterior compartment	0.0199
MFM Domain 2 score	0.0192
MMT - Shoulder Abduction	0.0162
Disease duration	0.0145
Time to descend 4 stairs - velocity	0.0145
HHM - Hip adduction	0.0141
MMT - Wrist Flexion	0.0136
MMT - Knee Flexion	0.0135
Teen exercise levels	0.0125
MMT - Hip Adduction	0.0112
Muscle biopsy WB	0.0089
MMT - Shoulder Flexion	0.0087
MMT - Ankle Dorsiflexion	0.0083
MMT - Hip Extension	0.0078
Muscle biopsy IHQ	0.0077
Gender	0.0075
CSA - Thigh medial compartment	0.0071
cCSA - Thigh posterior compartment	0.0057
MFM Total score	0.0054
HHM - Pinch grip	0.0051
HHM - Ankle dorsiflexion	0.0051
6MWT - Total distance	0.0048
NSAD Total Score	0.0039
FF - Lower leg posterolateral compartment	0.0038

Table S4 Mann-Whitney U test p-values comparing patients in the fast stage of progression vs those in the moderate stage of progression

**Baseline variables (Cross-sectional)**	**Fast stage of progression Me (IQR)**	**Moderate stage of progression Me (IQR)**	**p value**
Age at assessment (years)	29.0 (23.3 – 38.8)	35.0 (29.0 – 49.0)	<0.001
Disease duration (years)	8.0 (6.0 – 14.0)	14.0 (10.0 – 19.0)	<0.001
Creatine phosphokinase (U/L)	6630.0 (3577.0 – 9574.5)	3486.0 (2501.0 – 5310.5)	<0.001
HHM - Ankle plantar flexors (knee flexed) (lb)	11.5 (6.3 – 18.6)	15.5 (7.8 – 27.9)	<0.001
HHM - Ankle plantar flexors (knee straight) (lb)	12.9 (7.1 – 23.3)	17.5 (7.6 – 32.6)	<0.01
HHM – Grip (lb)	81.0 (47.0 – 112.0)	90.0 (61.0 – 129.0)	<0.01
HHM - Hip abduction (lb)	22.1 (15.8 – 30.0)	25.4 (17.0 – 35.2)	0.012
HHM - Hip adduction (lb)	13.8 (9.2 – 20.8)	16.5 (10.2 – 26.5)	0.012
HHM - Knee flexion (lb)	11.2 (5.4 – 24.5)	16.6 (7.5 – 28.4)	<0.001
HHM - Pinch grip (lb)	27.0 (16.5 – 37.0)	31.6 (23.0 – 41.0)	<0.001
HHM - Wrist flexion (lb)	18.3 (12.7 – 24.5)	21.0 (16.0 – 26.6)	<0.01
MMT - Ankle Eversion	6.0 (4.0 – 8.0)	7.5 (4.0 – 10.0)	<0.01
MMT - Ankle Inversion	7.0 (5.5 – 9.0)	8.5 (6.5 – 10.0)	<0.001
MMT - Ankle Plantarflexion (knee straight)	3.0 (2.0 – 7.0)	5.0 (2.0 – 10.0)	<0.001
MMT - Ankle Plantarflexion (knee flexed)	5.0 (3.0 – 7.0)	6.0 (3.0 – 9.0)	0.016
MMT - Elbow Extension	8.5 (7.0 – 9.5)	9.0 (7.0 – 10.0)	0.016
MMT - Elbow Flexion Brachioradialis	8.0 (7.0 – 10.0)	9.0 (8.0 – 10.0)	<0.001
MMT - Elbow Flexion Biceps	8.0 (7.0 – 9.5)	9.0 (7.5 – 10.0)	<0.001
MMT - Hip Adduction	3.0 (2.0 – 5.0)	4.0 (2.0 – 8.0)	<0.01
MMT - Hip Abduction	6.0 (3.0 – 8.0)	7.5 (5.5 – 9.0)	<0.001
MMT - Hip Extension	3.8 (3.0 – 6.4)	6.0 (3.0 – 7.5)	<0.001
MMT - Hip Flexion	6.5 (3.0 – 8.0)	7.5 (6.0 – 9.0)	<0.001
MMT - Knee Flexion	6.0 (3.0 – 7.0)	7.0 (4.0 – 8.0)	<0.001
MMT - Shoulder Abduction	7.0 (6.0 – 8.0)	7.5 (7.0 – 9.0)	<0.001
MMT - Shoulder Flexion	7.0 (6.0 – 8.0)	7.5 (6.5 – 9.0)	<0.01
MMT - Wrist Extension	9.3 (8.0 – 10.0)	10.0 (9.0 – 10.0)	<0.001
Time to run/walk 10 meters (s)	8.3 (7.0 – 10.6)	7.6 (4.9 – 10.6)	0.016
Time to run/walk 10 meters – velocity (m/s)	1.2 (0.9 – 1.4)	1.3 (0.9 – 2.0)	0.016
6MWT - Total distance (m)	365.0 (297.0 – 425.3)	385.0 (301.0 – 500.0)	0.048
6MWT – velocity (m/s)	1.0 (0.8 – 1.2)	1.1 (0.8 – 1.4)	0.017
Time to rise from floor (s)	5.7 (4.1 – 9.5)	4.6 (3.1 – 8.0)	<0.01

Time to rise from floor – velocity (m/s)	0.2 (0.1 – 0.2)	0.2 (0.1 – 0.3)	0.017
FF – Gracilis (%)	7.9 (6.1 – 12.3)	11.3 (6.5 – 21.6)	0.048
FF – Sartorius (%)	10.8 (7.6 – 16.3)	13.5 (6.5 – 21.6)	0.044
FF - Vastus intermedius (%)	17.8 (8.3 – 33.5)	39.5 (12.7 – 54.3)	<0.001
FF - Vastus medialis (%)	22.3 (11.9 – 33.3)	38.2 (12.5 – 50.7)	0.036
cCSA - Adductor magnus (cm^2)	739.0 (411.6 – 1096.9)	527.6 (330.5 – 818.6)	0.048
cCSA - Extensor digitorum (cm^2)	217.4 (168.0 – 252.2)	163.7 (115.5 – 196.7)	<0.001
cCSA – Gracilis (cm^2)	285.2 (205.4 – 419.8)	209.8 (149.3 – 263.9)	<0.001
cCSA – Sartorius (cm^2)	286.7 (208.3 – 351.5)	204.3 (138.9 – 319.5)	<0.01
cCSA - Tibialis posterior (cm^2)	295.7 (220.5 – 360.5)	246.4 (176.6 – 308.9)	0.023
cCSA - Vastus intermedius (cm^2)	1000.1 (723.0 – 1351.8)	563.8 (365.7 – 990.2)	<0.001
cCSA - Vastus lateralis (cm^2)	795.8 (573.9 – 1115.8)	545.2 (377.9 – 1135.8)	0.042
CSA - Extensor digitorum (cm^2)	247.8 (208.7 – 284.1)	191.8 (156.1 – 241.7)	<0.001
CSA – Gracilis (cm^2)	320.4 (248.5 – 447.2)	237.3 (189.0 – 303.8)	<0.001
CSA – Sartorius (cm^2)	329.4 (250.2 – 401.3)	248.2 (169.0 – 365.4)	0.020
CSA - Tibialis posterior (cm^2)	357.8 (280.3 – 433.7)	210.5 (260.0 – 371.1)	<0.01
CSA - Vastus intermedius (cm^2)	1252.2 (1097.7 – 1634.2)	1025.2 (769.6 – 1432.3)	<0.01
FF - Thigh anterior compartment (%)	22.7 (10.9 – 32.3)	38.4 (12.5 – 51.4)	0.016
FF - Thigh medial compartment (%)	15.2 (10.2 – 23.7)	26.1 (13.0 – 34.6)	<0.01
cCSA - Thigh anterior compartment (cm^2)	751.8 (571.0 – 1078.6)	502.3 (332.9 – 1023.1)	0.020
cCSA - Thigh medial compartment (cm^2)	452.6 (281.6 – 625.6)	336.6 (239.1 – 447.9)	<0.01
cCSA - Lower leg anterior compartment (cm^2)	262.2 (204.0 – 322.3)	211.5 (132.0 – 281.0)	<0.01
CSA - Lower leg anterior compartment (cm^2)	316.1 (270.8 – 366.5)	266.7 (196.5 – 328.3)	0.137
